# Visualizing Degradation of Cellulose Nanofibers by Acid Hydrolysis

**DOI:** 10.1021/acs.biomac.0c01625

**Published:** 2021-02-01

**Authors:** Panagiotis Spiliopoulos, Stefan Spirk, Timo Pääkkönen, Mira Viljanen, Kirsi Svedström, Leena Pitkänen, Muhammad Awais, Eero Kontturi

**Affiliations:** † Department of Bioproducts and Biosystems, School of Chemical Engineering, Aalto University, P.O Box 16300, Aalto 00076, Finland; ‡ Institute of Bioproducts and Paper Technology, Graz University of Technology, Graz 8010, Austria; § Department of Physics, University of Helsinki, P.O. Box 64, Helsinki FI-00014, Finland

**Keywords:** cellulose degradation, nanocellulose, order/disorder transitions, atomic force microscopy

## Abstract

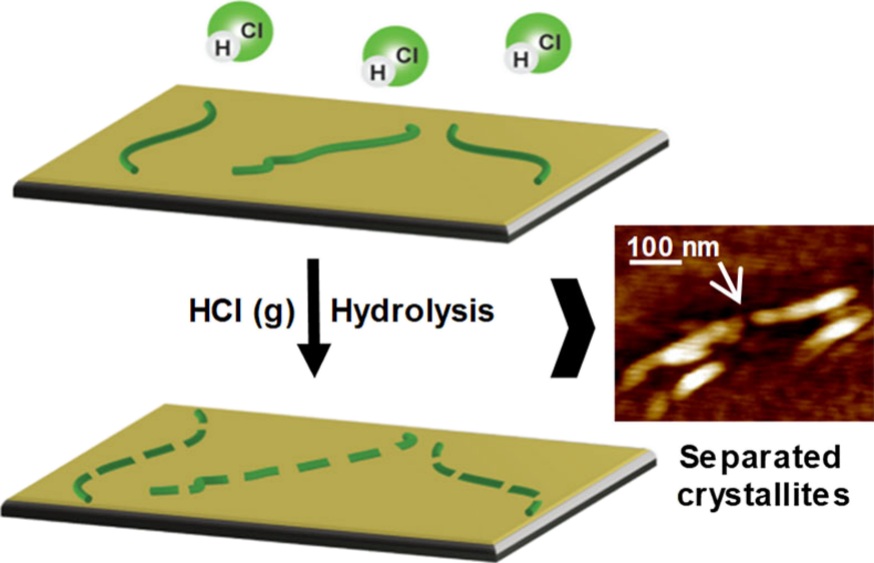

Cellulose hydrolysis is an extensively studied process due to its relevance in the fields of biofuels, chemicals production, and renewable nanomaterials. However, the direct visualization of the process accompanied with detailed scaling has not been reported because of the vast morphological alterations occurring in cellulosic fibers in typical heterogeneous (solid/liquid) hydrolytic systems. Here, we overcome this distraction by exposing hardwood cellulose nanofibers (CNFs) deposited on silica substrates to pressurized HCl gas in a solid/gas system and examine the changes in individual CNFs by atomic force microscopy (AFM). The results revealed that hydrolysis proceeds via an intermediate semi-fibrous stage before objects reminiscent of cellulose nanocrystals were formed. The length of the nanocrystal-like objects correlated well with molar mass, as analyzed by gel permeation chromatography, performed on CNF aerogels hydrolyzed under identical conditions. Meanwhile, X-ray diffraction showed a slight increase in crystallinity index as the hydrolysis proceeded. The results provide a modern visual complement to >100 years of research in cellulose degradation.

## Introduction

**Figure 1:**
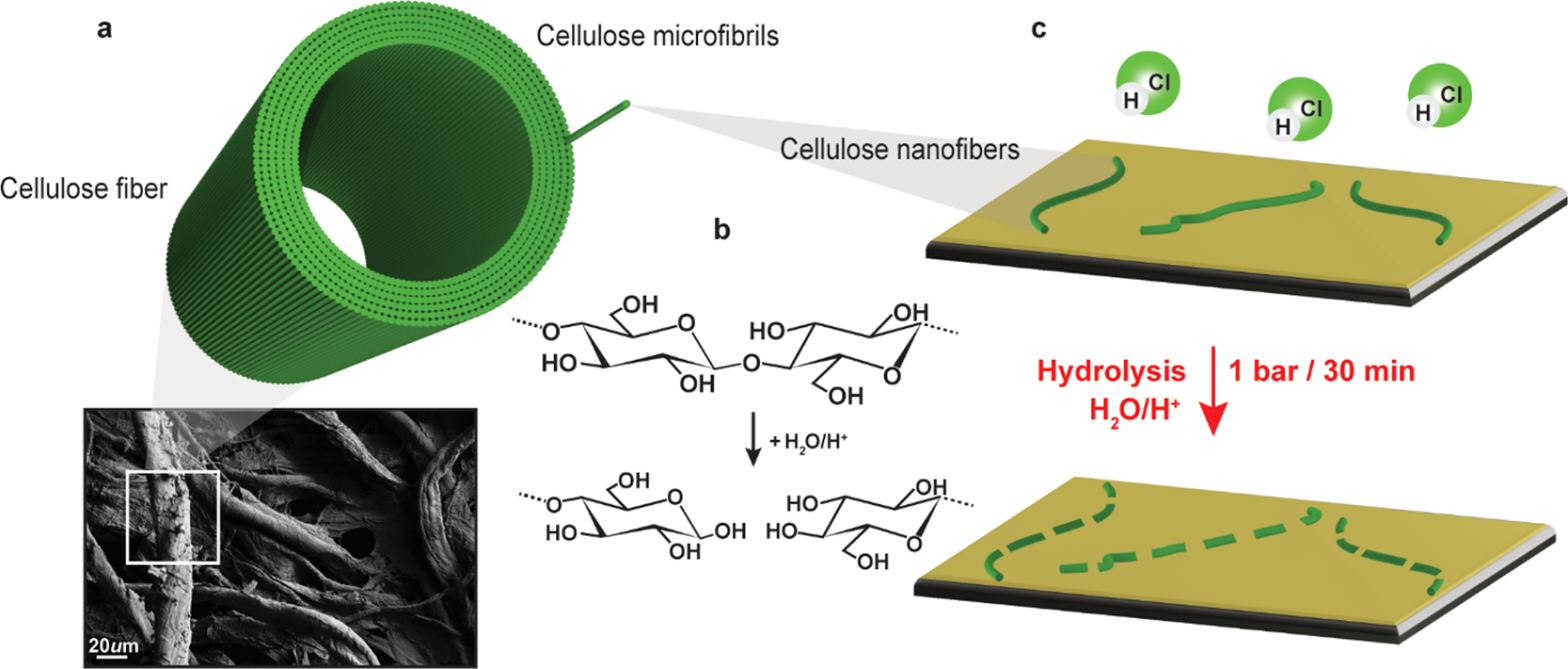
Cellulose fibers and their constituent subunit cellulose microfibrils (a), cellulose hydrolysis (b), individualized nanofibers on SiO_2_ substrate hydrolyzed via HCl (g) treatment and respective CNC analogue formation (c).

Cellulose (Figure 1a), the structural polysaccharide of the green plant cell wall,^1,2^ is characterized by very specific reactivity: its OH groups are generally less reactive than common alcohols,^3^ while it is infamously recalcitrant to degradation of its glycosidic bond.^4^ Concerning degradation, the semi-crystallinity of the basic supramolecular unit, that is, the cellulose microfibril (CMF) is a key issue because the short disordered, non-crystalline regions are much more susceptible to degradation than the long crystalline counterparts. This gives rise to the so-called leveling-off degree of polymerization (LODP), which is reached most notably in acid hydrolysis of cellulose (Figure 1c), where after a rapid phase of chain cleavage in the disordered regions, the degradation nearly halts, hitting the LODP when only the crystalline segments are left.^5^ On a wider perspective, degradation carries increased industrial potential: full hydrolysis to glucose bears significance in biofuel fabrication^6,7^ —though not usually through the acid hydrolysis route— and industrial sites are emerging for cellulose nanocrystal (CNC) preparation, which is essentially based on reaching the LODP.^8,9^

Hydrolysis of cellulose, that is, the addition of acidic water to yield glucose from anhydroglucose (Figure 1b) is usually performed in a heterogeneous liquid/solid system,^10,11^ consisting of, for example, solid fibers immersed in an aqueous medium. The connection between the semi-crystallinity of the native CMF and acid hydrolysis is well established, but a direct visual link is missing. In fact, no one has ever really seen the disordered regions in a CMF because they are allegedly very short, just 4–5 anhydroglucose units (∼1–2 nm) in length,^5^ making many schematic representations, in which the CMFs are depicted as possessing bulky “amorphous” segments, inaccurate. When fibers are hydrolyzed in a liquid/solid medium, their morphology is significantly altered, rendering it difficult to make any systematic visual observations in nanoscale. Although atomic force microscopy (AFM) has been applied to observe enzymatic hydrolysis in situ in the nanoscale,^12−14^ acquisition of visual data on crystalline/disordered alterations upon the hydrolysis has not been feasible with this approach. To circumvent these complications, we have utilized a gas/solid system with HCl gas and isolated CMFs in the form of cellulose nanofibers (CNFs), immobilized on a flat 2D substrate to investigate the morphological intricacies of the degradation ex situ by AFM (Figure 1c). Previously, we have shown that besides the degradation, hydrolysis with HCl (g) leaves the morphology of cellulosic fibers largely intact.^5,15^ Thereby, we based this study on a hypothesis that individual CNFs on a surface would show cleavage from their dislocations (Figure 1c), enabling us to draw correlations between the visual evidence and degree of polymerization (DP) data, extracted with gel permeation chromatography (GPC) from bulk hydrolysis of CNF aerogels. The results provided unprecedented visual data on the nature and degradation behavior of natural polymers. While rearrangements in solid–gas reactions have been reported also in situ,^16^ degradation has mainly been visualized on synthetic polymers upon, for example, thermal annealing^17^ or fatal adsorption.^18^

## Experimental Section

### Materials

Millipore water of 18.2 MΩ·cm resistivity at 25 °C was used. A CNF dispersion was employed (isolated from bleached hardwood kraft pulp/6 times fluidized), prepared according to Eronen et al.^19^ CNF composition was determined via high-performance anion exchange chromatography pulse amperometric detection (HPAEC-PAD) and its hemicellulose content was ca. 24% (23.5% xylose, 0.5% mannose, see Figure S1 in the Supporting Information; note that HPAEC is not capable of distinguishing the small amount of methyl glucuronic acids from cellulose-based glucose). This composition is expected for hardwood kraft samples and in agreement with previous research.^20^ The HCl (g) bottle (99.8%, 10 dm^3^, 6 kg) was purchased from AGA (Sweden). Silicon wafers (Si 100) were acquired from Okmetic, Espoo (Finland).

### Substrates

CNF thin films were prepared by spin-coating on SiO_2_ freshly cleaved substrates. The substrates were cleaned through Milli-Q water and acetone, while ozonation through an UV.TC.EU.003 ozone cleaner (Bioforce Nanoscience, Utah, USA) for 25 min took place.

### Spin-Coating

CNF (1 g/L) dispersion was spin-coated (4000 rpm, 90 s) through a Laurell Technologies WS-650SX-6NPP/LITE model, on cleaned SiO_2_ substrates.

### Freeze-Drying

CNF aerogels (1 g/L, 50 mL) were prepared through freeze-drying by a Freezone 2.5 instrument (Labconco, Kansas, USA) for 2 days (0.2 mbar, −47 °C).

### Acid Hydrolysis

The hydrolysis was performed in a custom-built reactor,^15^ while the pressure values were adjusted at 0.2, 0.6, and 1.0 bar. The hydrolysis time was set at 30 min, while overnight exposures also took place.

### Atomic Force Microscopy (AFM)

The hydrolyzed CNF films were examined by an AFM Multimode 8 microscope (E scanner) from Bruker AXS Inc. (Madison, Wisconsin, USA). The imaging was done with Ultrasharp μmarch silicon tips (HQ: NSC15/Al BS, Tallinn, Estonia) via the tapping mode. The typical force constant was 40 N/m, and the resonance frequency was 325 kHz. The particle widths were determined from the height scale in order to avoid the error caused by AFM tip convolution. The analysis of particle dimensions was subsequently done for the AFM images using ImageJ software.

### Gel Permeation Chromatography (GPC)

All samples were activated through the addition of water, acetone, and DMAc, followed by dissolution in 90 g/L LiCl in DMAc under magnetic stirring. The dissolved samples were diluted 10-fold, followed by filtering through a 0.2 μm syringe. To measure the molar mass distribution, a Dionex Ultimate 3000 (Sunnyvale, California, USA) instrument was used, with four Agilent PL-gel MIXED-A columns and a guard column (Santa Clara, USA). A Viscotek/Malvern SEC/MALS 20 multiangle light-scattering (MALS) detector and a Shodex differential index detector (DRI, Showa Denko, Ogimachi, Japan) were employed, while the flow rate was 0.75 mL/min. **The** injection volume was set at 100 μL. Detector constants (MALS and DRI) were determined using a narrow polystyrene sample (*M*_w_ = 96,000 g/mol, *Đ* = 1.04) dissolved in 0.9% LiCl in DMAc. A broad polystyrene sample (*M*_w_ = 248,000 g/mol, *Đ* = 1.73) was used for checking the detector calibration. The ∂*n*/∂*c* value of 0.136 mL/g was used for celluloses in 0.9% LiCl in DMAc, as described by Potthast et al.^21^

### X-ray Diffraction (XRD)

The wide-angle X-ray scattering (WAXS) measurements were conducted with a custom-built X-ray scattering setup at the Department of Physics, University of Helsinki. The X-rays were produced using a conventional X-ray tube with 36 kV voltage and 25 mA current from which the desired Cu-K_α_ radiation (wavelength λ = 0.154 nm) was selected using a Montel monochromator. The measurements were performed in the perpendicular transmission mode with a measurement time of 42 min, and the scattering data were collected onto a MAR345 image plate detector (Marresearch Norderstedt, Germany). The cellulose samples were measured, freeze-dried, and sealed between thin (*d* = 2.5 μm) Mylar foils in aluminum washers. Calibration of the scattering angle (2θ) was conducted using a lanthanum hexaboride powder sample. A 50° wide sector from the data was integrated and corrected for absorption, polarization, and scattering by Mylar and air. A semi-transparent beam stop was used to acquire the transmission values of the X-ray beam to be used for the absorption correction. The relative crystallinity index (Crl) was acquired from the scattering data for the measured samples. The crystallinity index was computed using an amorphous fitting method, described by Ahvenainen et al.^22^ In this method, the crystalline and amorphous components of the samples are modelled and then fitted to the experimental data into a chosen scattering angle range, which, in this case, was chosen to be 11–49°. The crystalline component was constructed using 18 reflections of cellulose *I*_β_, adopted from Nishiyama et al.^23,24^ The reflections were modelled as Gaussian functions, with their positions, widths, and heights fitted similarly as described by Ahvenainen et al.^22^ The amorphous contribution was approximated from an experimental scattering curve of a lignin sample, proven to be suitable for various materials.^22,25,26^

## Results and Discussion

### Formation, Morphology, and Dimensional Analysis of Degraded CNFs

**Figure 2:**
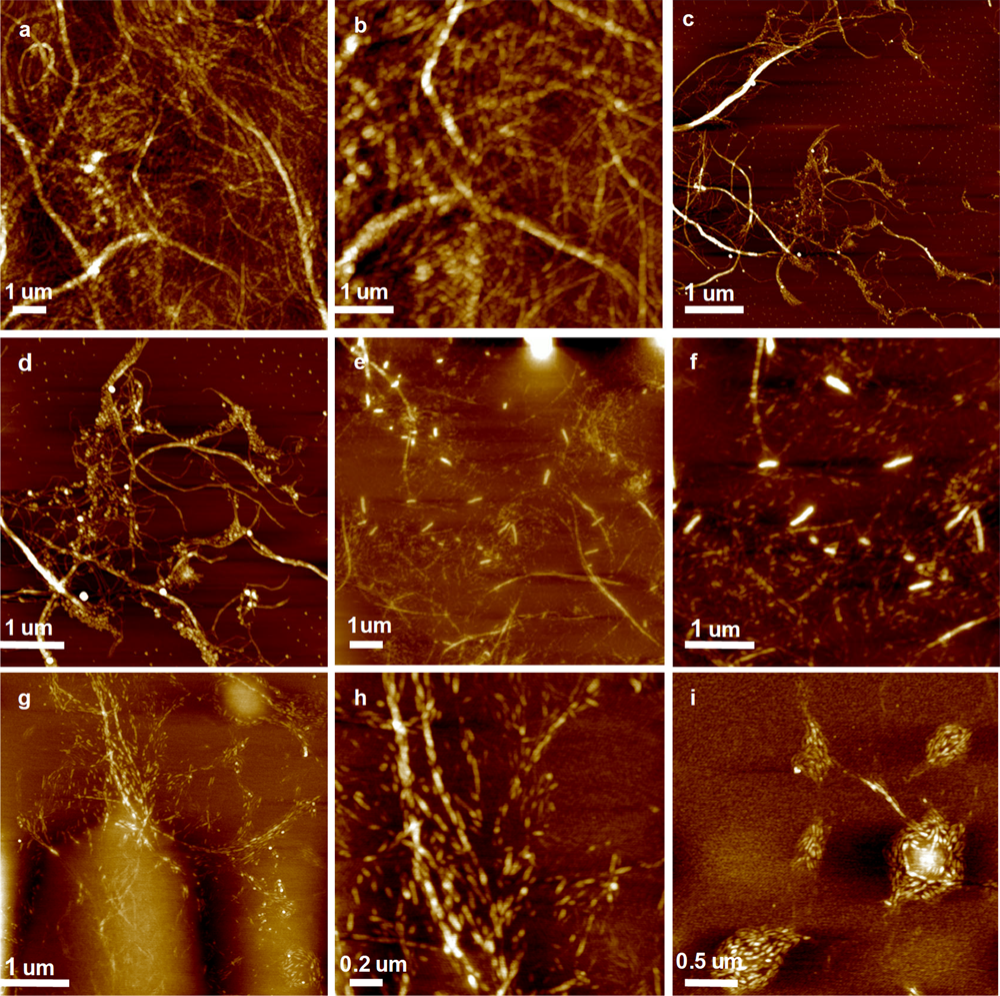
AFM height images of (a, b) CNF spin-coated on SiO_2_; (c, d) CNF after 0.2 bar HCl for 30 min; (e, f) CNF after 0.6 bar HCl for 30 min; and (g–i) CNF after 1.0 bar HCl for 30 min.

Figure 2 shows AFM images of the CNF thin films hydrolyzed under incremental HCl pressures at room temperature. Figure 2a,b demonstrates the CNF network as deposited upon spin-coating, providing close to full coverage over the substrate. When treated under mild HCl pressure conditions (0.2 bar, Figure 2c,d), the CNFs did not exhibit any morphological alterations. By contrast, an increase in the HCl pressure to 0.6 bar (Figure 2e,f) led to explicit degradation of the CNFs as they were transformed into conspicuous nanorods, clearly shorter than the original CNFs. Importantly, however, the original shape of the CNF can still be discerned from the nanorod formulation.

As expected, increasing the HCl pressure further to 1.0 bar facilitated the degradation to a greater extent (Figure 2g–i) than at lower pressures. Overall, the degradation products (nanorods) were reminiscent of cellulose nanocrystals (CNCs), which is logical as CNCs are generally considered products of acid hydrolysis of native cellulose down to the LODP level. Meanwhile, the formation of disk-like assemblies from these CNC analogues was also observed on some parts of the silicon wafer after 1.0 bar HCl (g) exposure (Figure 2i). Curiously, after longer exposure times to higher HCl vapor pressure, only disk-like aggregates of cellulose crystallites were to be found (Supporting Information, Figure S2). Experiments with pure silicon wafers suggested that the disk-like patterns occurred due to the chemical and morphological changes of the Si/SiO_2_ substrate under elevated HCl pressures (Figure S4). To further illuminate the role of the Si/SiO_2_ substrate with HCl, submonolayers of readily prepared CNCs (by sulfuric acid hydrolysis from cotton linters) were subjected to HCl (g) at 1.0 bar and similar disk-like agglomerates were found as in Figure 2i (Figure S3, Supporting Information). These results suggest that a reaction between SiO_2_ and high-pressure HCl under long exposures causes distinct morphological changes in SiO_2_ overlayer on the silicon wafer, resulting in the migration of the formed CNC analogues into circular, disk-like aggregates as indicated in Figure S4 (Supporting Information). The exact compositional alteration in the Si/SiO_2_ substrate, however, could not be established (see Figure S4, Supporting Information). In any case, the migration of the hydrolyzed particles on the silica substrate accompanied by compositional alteration of the substrate itself was the reason why harsher conditions and/or longer exposures to gaseous HCl than at 1 bar for 30 min were not probed in this study.

**Figure 3:**
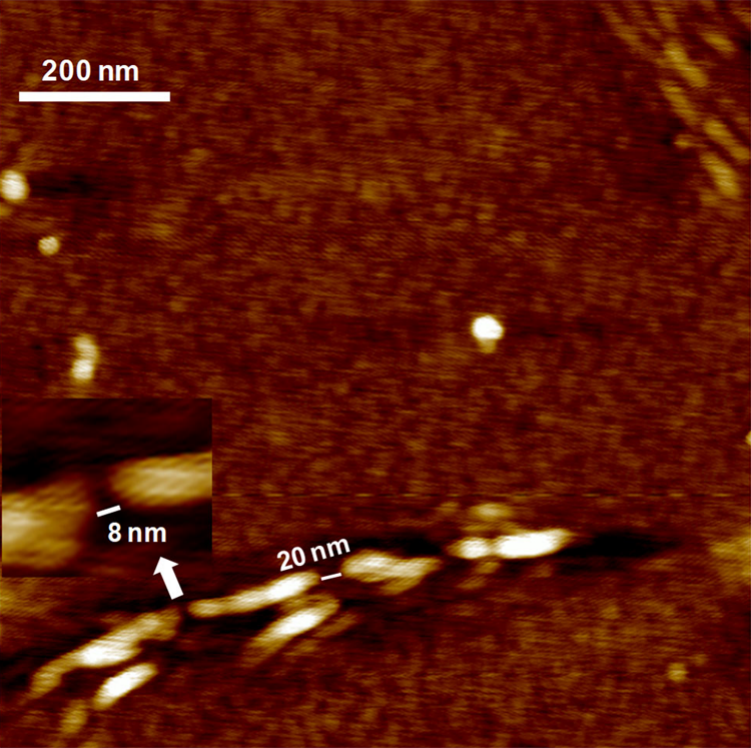
High-resolution 1 × 1 μm^2^ AFM height image of hydrolyzed CNFs formed after 1.0 bar HCl (g) pressure applied for 30 min.

For a more precise morphological analysis of the CNC analogue formation, Figure 3 presents an AFM image of increased resolution of the CNF sample hydrolyzed at 1.0 bar HCl (g). An ostensible explanation of the events in Figures 2 and 3 lies in the fringed-fibrillar model: noncrystalline segments are hydrolyzed as the crystallites remain more or less intact. However, the hydrolysis by HCl (g) does not involve mass transfer apart from the adsorption of HCl on CNFs before the hydrolysis and consequent desorption afterward. Indeed, there has been no previously observed discernible change in the morphology of micrometer-sized cellulosic fibers after HCl (g) hydrolysis, not even in the nanoscale resolution.^27^ The removal of dissolving sugar units via rinsing after the hydrolysis could perhaps reveal gaps between the crystallites, but rinsing has not been applied here as it would inevitably cause morphological alterations that suppress the original alterations that are due to the hydrolysis. The emergence of the gaps may hypothetically be caused by a slight movement of the crystallites on the Si/SiO_2_ substrate, which ultimately results in the formation of the disk-like aggregates under longer exposures (Figure 2i). In addition, as revealed from Figure 3, the gap lengths between the crystallites are far exceeding that of disordered regions,^5^ further strengthening the hypothesis on crystallites movement with respect to each other. However, statistical quantification on the gap spacings would be misleading because the AFM tip convolution effect leads to underestimation of the gap length. In addition, it is unclear how xylan on the CNFs influenced the morphology of the system upon hydrolysis. We emphasize that although xylan degrades more readily than cellulose, the residues of its degradation are not removed during the hydrolysis because no rinsing by water was applied on the surfaces afterward. Whether xylan affects the actual cellulose hydrolysis is discussed in more detail with the GPC data below.

**Figure 4:**
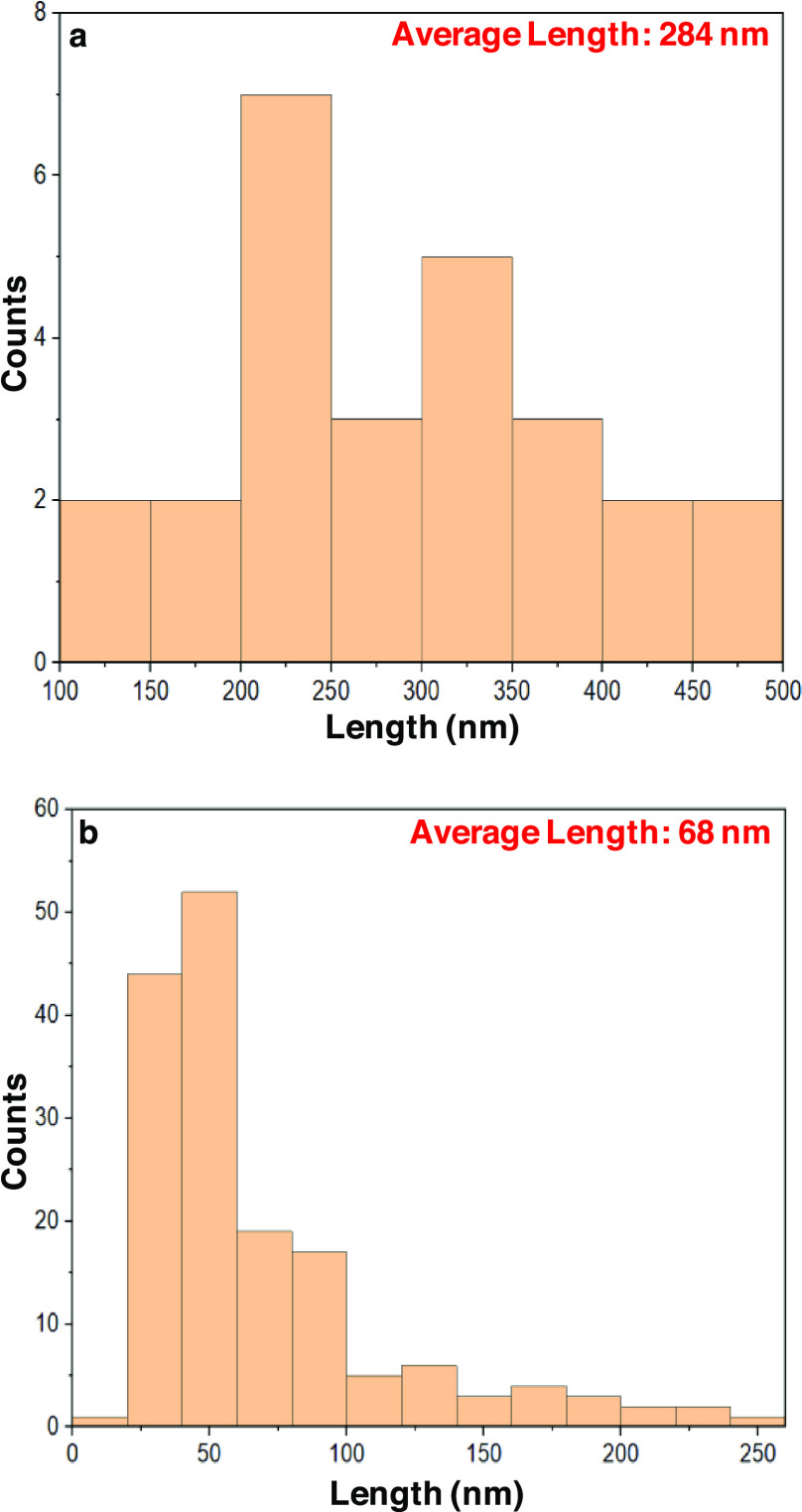
Length distribution histograms for the CNF films hydrolyzed at 0.6 bar HCl (g) (standard deviation 90 nm) (a) and 1.0 bar HCl (g) (standard deviation 46 nm) (b).

To find out about how the dimensions of the formed particles correspond to the hydrolysis, length distribution histograms were constructed from Figure 2h,f for 0.6 bar and 1.0 bar of HCl (g) pressure treatments, respectively (Figure 4). The longer particles after 0.6 bar HCl (g) hydrolysis (Figure 4a) are reasonably expected, corresponding to an intermediate stage between whole CNFs and CNC analogue formation. On the contrary, for the films hydrolyzed at 1.0 bar HCl (g), the average particle length was 68 nm (Figure 4b), exhibiting a relatively good agreement for CNCs based on chemical wood pulp in comparison to the literature,^28−31^ which strengthens the validity of the demonstrated results. After all, the actual novelty of this study focuses on visualization of the hydrolysis phenomenon itself, utilizing 2-dimensional model CNF films in an attempt of providing a visual linkage between semi crystallinity and gas-phase induced hydrolysis.

**Figure 5:**
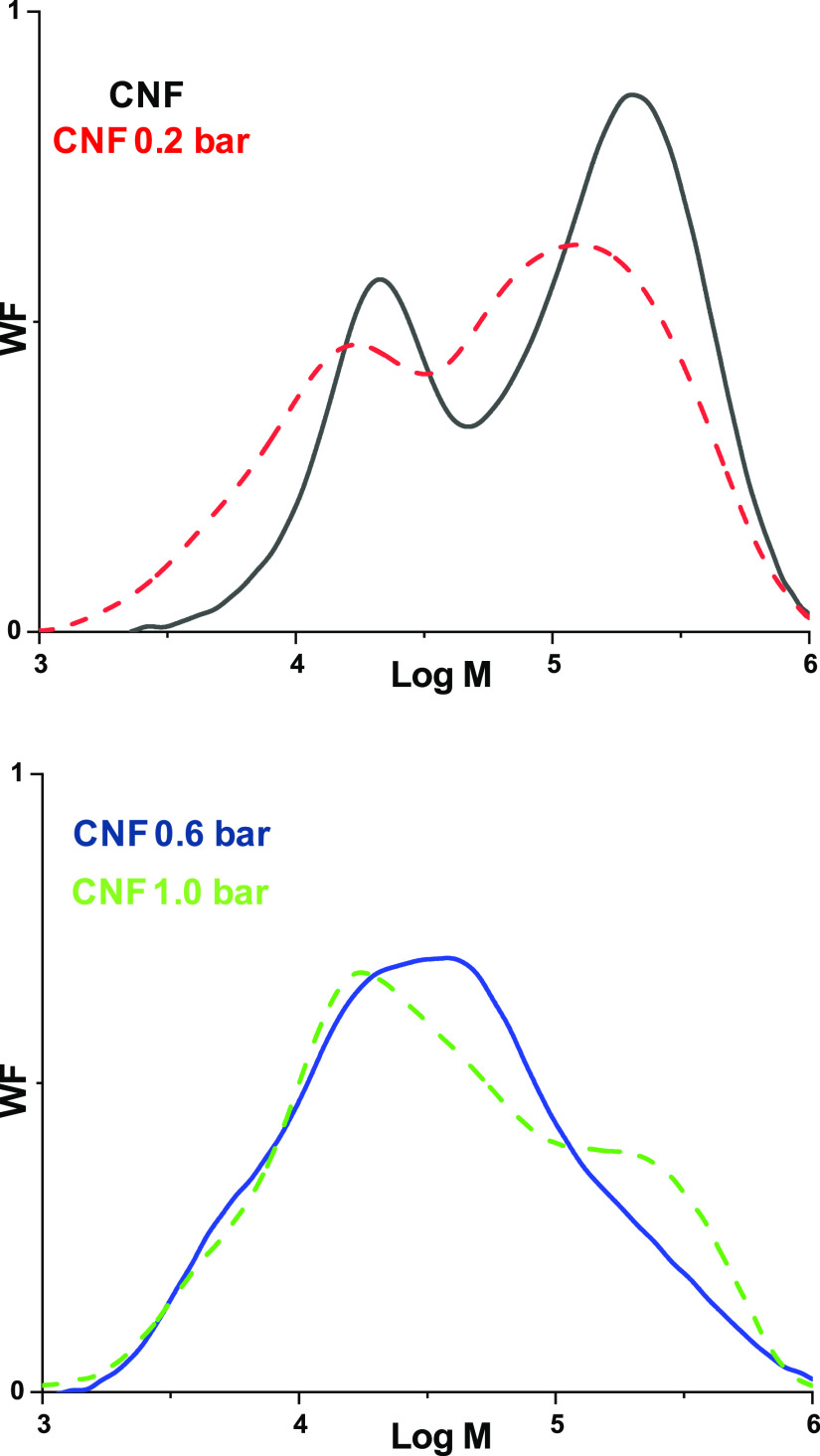
Molar mass (M) distribution curves for the CNF and CNF 0.2 bar HCl (g) samples (a) and CNF 0.6 bar and 1.0 bar (b)

To investigate what really happens to the CNF when they are exposed to HCl (g) and to link it with the visual evidence from AFM, the development of molar mass (M) was followed. As the sample amount on silicon wafers would not be sufficient, CNF aerogels prepared through freeze-drying were treated with HCl (g) under the same set of pressures as the CNF films and further examined with GPC (Figure 5).

Figure 5a demonstrates a bimodal distribution for untreated CNF from birch kraft pulp: the low M fraction is mainly assigned to xylan, while the high M fraction is affiliated with cellulose, as already established in the literature.^32−34^ However, as the hydrolysis proceeds, the identification between the peak (M fraction) and cellulose/xylan becomes vague. Several accounts have shown that xylan impedes cellulose hydrolysis when incorporated in the system.^32−35^ Håkansson et al.^33^ have speculated that the tight binding of xylan to cellulose may partly protect both xylan and cellulose from hydrolysis. All in all, the simultaneous hydrolysis of xylan and cellulose together with any possible protective effect of xylan, renders direct peak assignment to either xylan or cellulose fraction impossible during the hydrolysis.

More intense hydrolytic conditions increase the ratio of the low M contribution in the whole distribution from 0.1 (CNF) to 0.3 for the sample hydrolyzed at 1.0 bar of HCl pressure, while the polydispersity index (*Đ*_M_) is increasing through hydrolysis, since particles of diverse molecular weight are created. Table 1 presents the values extracted from GPC for all samples.

**Table 1:** Weight Average Molecular Weight (***M***_W_), Number Average Molecular Weight (***M***_n_), Degree of Polymerization (DP) and Polydispersity Index (*Đ*_M_) Values of CNF, CNF 0.2 Bar, CNF 0.6 Bar and CNF 1.0 Bar in HCl (g) for 30 min, Extracted Out of the Corresponding M Distribution Curves from GPC

sample	*M*_W_	*M*_n_	DP	*Đ*_M_
CNF	164,078	41,346	1012	4.0
CNF 0.2 bar	122,230	20,922	754	5.8
CNF 0.6 bar	78,403	16,696	483	4.7
CNF 1.0 bar	91,269	16,082	563	5.7

Rather than looking at the average *M*_W_ values in Table 1, the full distributions in Figure 5 are far more informative. The bimodal M distribution curves reveal that even after HCl (g) treatment under 1.0 bar of pressure, unhydrolyzed high M fragments still remain, corresponding to the high M region of the curve. The AFM image (Figure 2g) obtained directly from the corresponding CNF film is in line with the GPC data of Figure 5, as co-existing rod-like CNC analogues (low *M*) and unhydrolyzed fragments (high *M*) can still be observed. It is reasonable to assume that after HCl (g) treatment at 1.0 bar, both the low *M* and the high *M* contribution mainly arises from hydrolyzed segments of cellulose, as most of the xylan content has been degraded to monomers or oligomers. Prolonging the hydrolysis time to overnight exposure leads to a further increase of the low *M* contribution to 0.6 (Figure S5, Supporting Information) with an average DP value of 377. No qualitative difference in the distribution shape can be observed. In fact, even after overnight hydrolysis at the extensive pressure of 1.0 bar, the LODP has still not been reached, as most previous works^32−34^ indicate lower values for hardwood samples. It appears that such recalcitrance can be assigned to the initially high xylan content.

**Figure 6:**
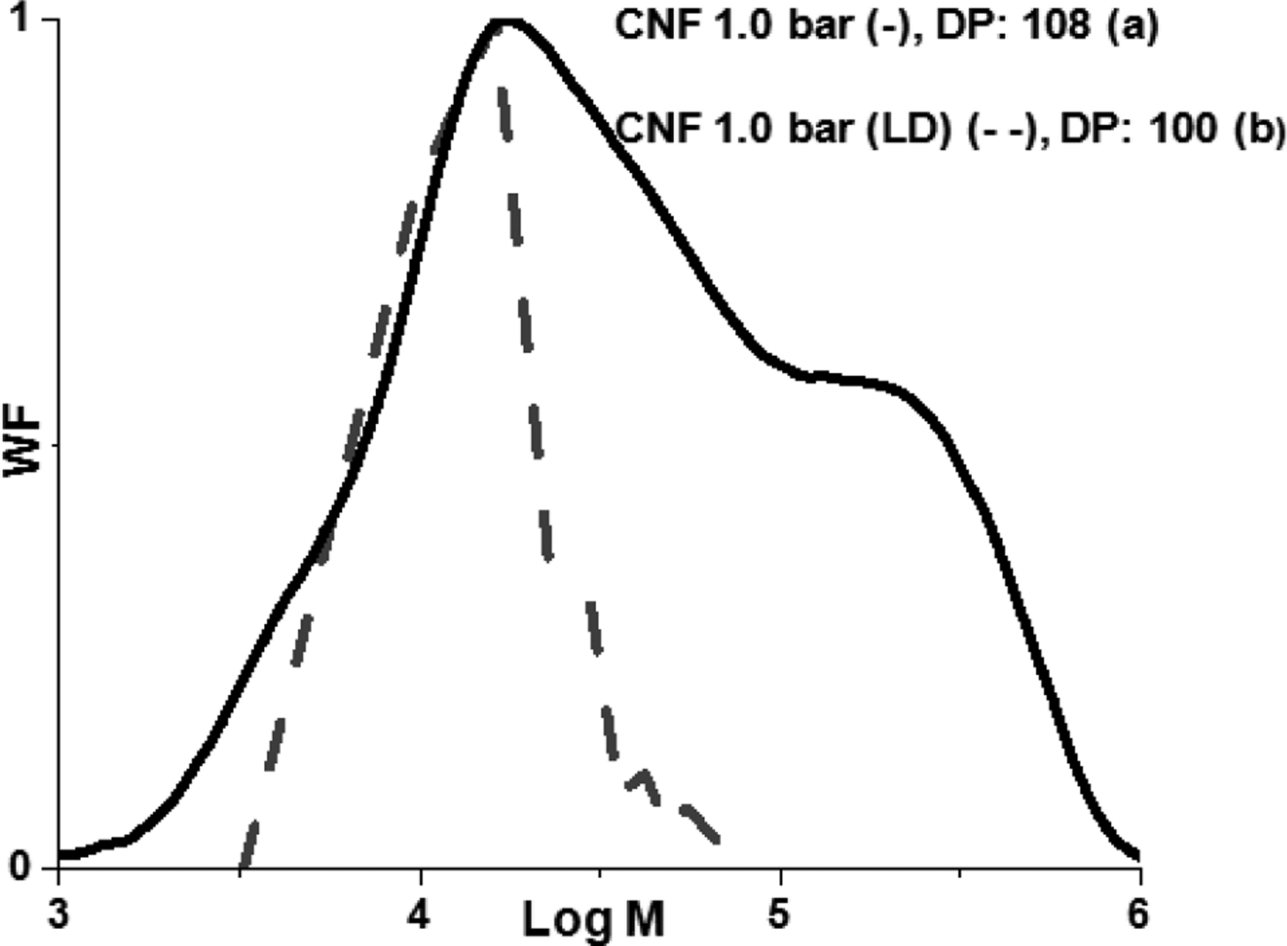
M distribution for the CNF after 1.0 bar HCl (g) hydrolysis (continuous line, a) and M distribution for the same sample extracted from the length distribution (LD) histograms constructed from AFM image (dashed line, b).

Given the cellulose I unit cell parameters,^24^ a DP of 2 in the cellulose crystal corresponds to a length of 1.013 nm. With this correlation, the length values of Figure 4b were converted into (logarithmic) M data (see more detailed calculations in the Supporting Information) and compared to the genuine (logarithmic) M data by GPC (Figure 6). There is a very good correlation between the length distribution from the AFM and the low M fraction of the GPC after 30 min 1.0 bar hydrolysis. Therefore, it seems plausible that the CNC analogues in Figure 2g–i are indeed cellulose crystallites, hydrolyzed down to the LODP.

A fine convergence can be observed, as the DP value of 108 corresponding to the peak position of low M region of GPC data and the 100 extracted from the LD distribution peak, exhibit quite low divergence. These values are quite representative for hardwood samples hydrolyzed down to LODP,^20,33−35^ which indicates that the low M region of Figure 6 arises only due to crystalline cellulose contribution. In addition, the DP of 108 corresponds to a length value 55 nm, not far from the 68 nm value provided by the average of the length histogram (Figure 4b). After all, nice agreement between the AFM and GPC data could be pointed out, considering also that the tip convolution effect^36^ can lead to overestimation of the crystallite dimensions.

### Crystallinity Data

XRD patterns for dried CNF aerogels are demonstrated in Figure S6 (Supporting Information), while Table 2 presents the crystallinity index (CrI), as calculated through the corresponding diffraction peaks. The increase in crystallinity upon HCl (g) hydrolysis under 0.2 and 0.6 bar for 30 min was negligible. We again emphasize that HCl (g) treatment does not remove hemicelluloses from the aerogel structure, but merely degrades them into shorter, likely amorphous, units^37^ which actually do remain on the fibrillar structure after hydrolysis. From this perspective, the negligible crystallinity alteration after mild HCl (g) pressure treatment can be excused. However, a 20% increase in the crystallinity index, from 0.35 to 0.42, is observed after hydrolysis at 1.0 bar of pressure, in line with previous reports on HCl (g) hydrolysis of cellulose.^27^ The presence of the nonfreezing bound water layer—corresponding to tightly bound water on the cellulosic surface^38−40^ —which does not exceed 4 wt %, renders the system as only partially hydrated allowing at the same time HCl (g) molecules dissociation. Kontturi et al.^27^ have discussed that partially or nonhydrated (solid/gas) systems favor crystallization over fully hydrated aqueous (solid/liquid) systems due to the lower heat of crystallization, decreasing the thermodynamic barrier for the transition. The crystallization is only notable when significant portions of cellulose have been hydrolyzed, that is, with the sample treated at 1.0 bar HCl (g). Finally, insignificant alteration in the 200 peak position or the crystallite size calculated from the FWHM value of the XRD peak was induced through HCl (g) treatment, as demonstrated in Table S1 in the Supporting Information. Because of the polydispersity of the CNF height in AFM analysis, the crystallite size, deduced from XRD data, appears a more reliable measure of unchanged crystallite during the hydrolysis than AFM height profiles for CNF (Figure S7).

**Table 2:** Crystallinity Index (CrI) for CNF Aerogels, Treated in Varying HCl (g) Pressures for 30 min^a^

sample	CrI
CNF	0.35
CNF 0.2 bar	0.36
CNF 0.6 bar	0.37
CNF 1.0 bar	0.42

a The Experimental Error Was Calculated to be 0.03 for all Measured Values

## Conclusions

Degradation and crystallization of cellulose was visualized via the exposure of CNF thin films on the HCl (g) pressure. The coexistence of hydrolyzed and unhydrolyzed fragments was observed, which was in good agreement with the GPC data of aerogels prepared from the same CNF batch. The low molar mass fraction of the hydrolyzed samples was in nice conformity with the LODP of wood-based cellulose substrates, while dimensional analysis of CNC analogues obtained from hydrolysis displayed good agreement with the M data. Finally, exposure of the aerogels on hydrogen chloride gas led to crystallization of the amorphous cellulose regions, increasing the crystallinity index of the samples.

## Supporting Information Available

HPAEC results, additional AFM images, additional GPC data, X-ray diffraction patterns, additional calculations (PDF)
