# Supplementary material for: Visualizing Degradation of Cellulose Nanofibers by Acid Hydrolysis
Source: Biomacromolecules. 2021 Feb 1;22(4):1399–405. doi: 10.1021/acs.biomac.0c01625 (PMC8045026; doi:10.1021/acs.biomac.0c01625)
Supplement: Supplementary file 1 — bm0c01625_si_001.pdf [file bm0c01625_si_001.pdf]

Supporting Information

Visualizing Degradation of Cellulose Nanofibers by Acid Hydrolysis

Panagiotis Spiliopoulos[†], *Stefan Spirk*[‡], *Timo Pääkkönen*[†], *Mira Viljanen*[§], *Kirsi Svedström*[§], *Leena Pitkänen*[†], *Muhammad Awais*[†], *Eero Kontturi*[†]*

† Department of Bioproducts and Biosystems, Aalto University, School of Chemical Engineering, P.O Box 16300, 00076, Aalto, Finland

‡ Institute of Bioproducts and Paper Technology, Graz University of Technology, Graz, Austria

§Department of Physics, University of Helsinki, P.O. Box 64, FI-00014 Helsinki, Finland

Table of contents

1 Analyzed data and images S3
1.1 High Performance Anion Exchange Chromatography (HPAEC) S3
1.2 Atomic Force Microscopy (AFM) S4
1.3 Gel Permeation Chromatography (GPC) S7
1.4 X-Ray Diffraction Pattern (XRD) S8
1.5 Height Profile Images S9
2 Additional Calculations S10
References S11

1. Analyzed Data and Images

1.1 High Perfomance Anion Exchange Chromatography (HPAEC)

The carbohydrate composition of the cellulosic sample was determined by quantitative saccharification upon acid hydrolysis, according to the standard procedure reported by Sluiter et al.[1] The monosaccharides were determined by high performance anion exchange chromatography with pulse amperometric detection (HPAEC-PAD) in a Dionex ICS-3000 system (Sunnyvale (CA), USA). Carbohydrate percentages were calculated from the monosaccharide contents using a correction factor.[1]

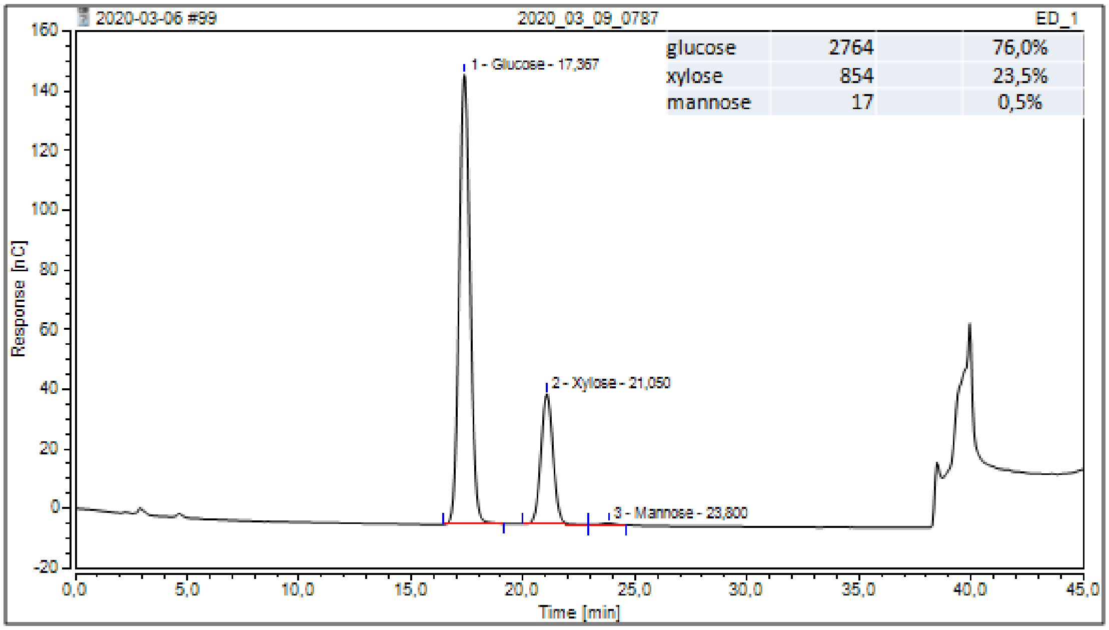

Figure S1. Chromatogram from HPAEC analysis after total hydrolysis of the CNF used. The carbohydrate composition is in agreement with previous results on hardwood kraft pulp samples.[2] The presence of glucuronic acid, present for most softwood and hardwood species[3], cannot not be distinguished from glucose via the HPAEC technique.

1.2 Atomic Force Microscopy (AFM)

Cellulose nanocrystals were produced from Whatman 1 cotton filter paper, via sulfuric acid hydrolysis (64 wt% H_2SO_4, 45 ^{o}C for 45 min.), as described by Edgar et al.[4] and further spin-coated (100 mg/l) on SiO_2 substrates before HCl (g) treatment.

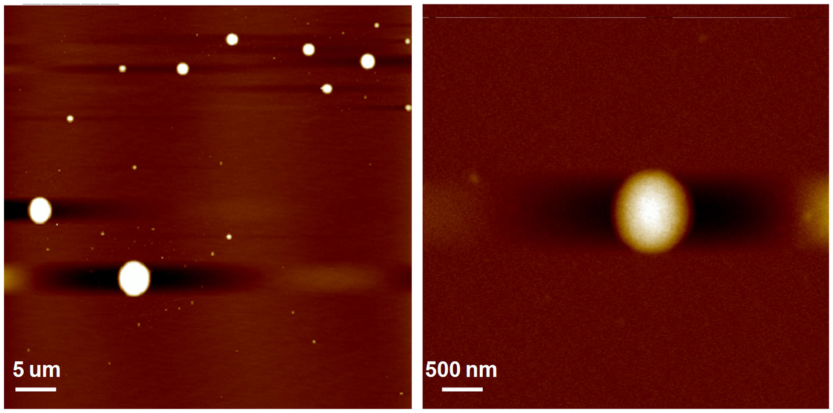

Figure S2. AFM images of CNF thin films on SiO_2 after 1.0 bar – 60 min of HCl (g) treatment.

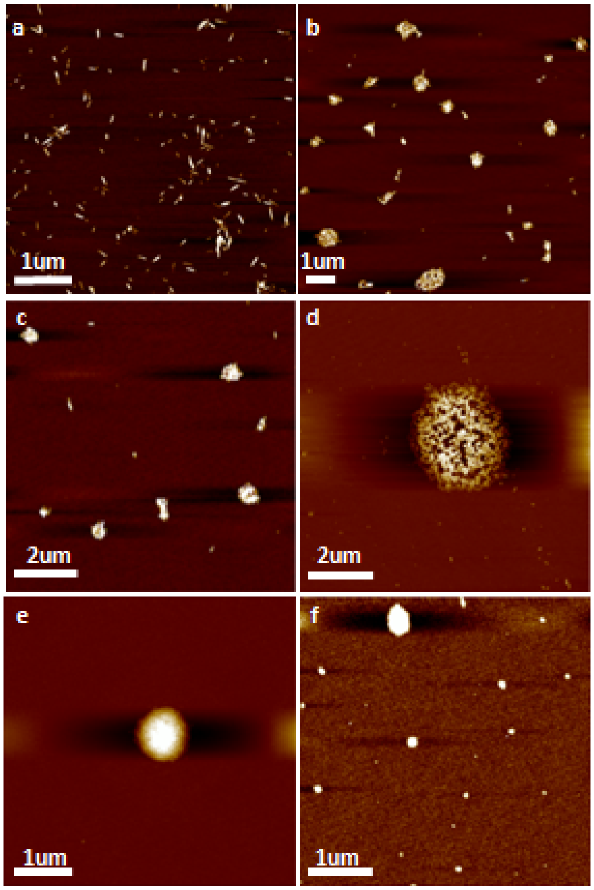

Figure S3. AFM images of CNC thin films after HCl (g) treatment (a) no HCl treatment, (b) 0.2 bar - 30 min, (c) 0.4 bar - 30 min, (d) 0.6 bar-30 min, (e) 0.8 bar - 30min, (f) 1.0 bar – 30 min.

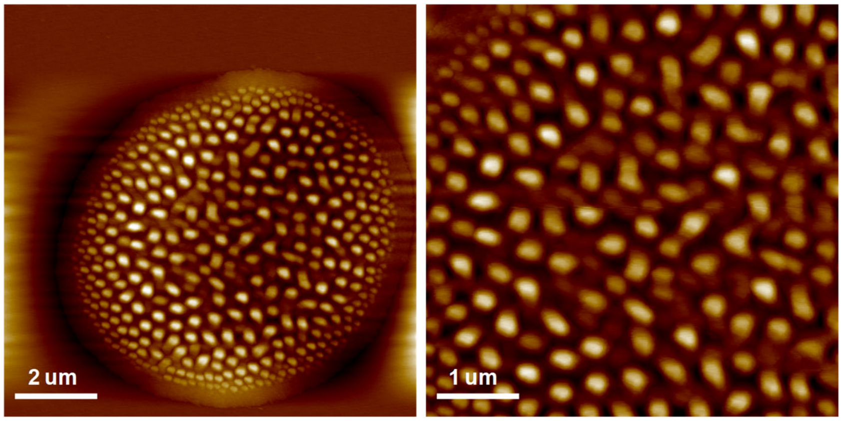

Figure S4. AFM images of the SiO_2 substrate after 1.0 bar - 30 min HCl (g) treatment. The interaction between SiO_2 and HCl (g) under increased pressure leads to a corruption of the initial substrate.

As observed from Figure S4, exposure of bare SiO_2 wafers to 1.0 bar – 30 min HCl (g) treatment, leads in alteration of the initial substrate morphology, as clearly discernible particles of spherical conformation can be observed. However, XPS analysis of the treated samples did not manage to provide an enlightening insight on the compositional alteration of the substrate. As a result, the exact type of interaction between HCl (g) and SiO_2 could not be established.

1.3 Gel Permeation Chromatography (GPC)

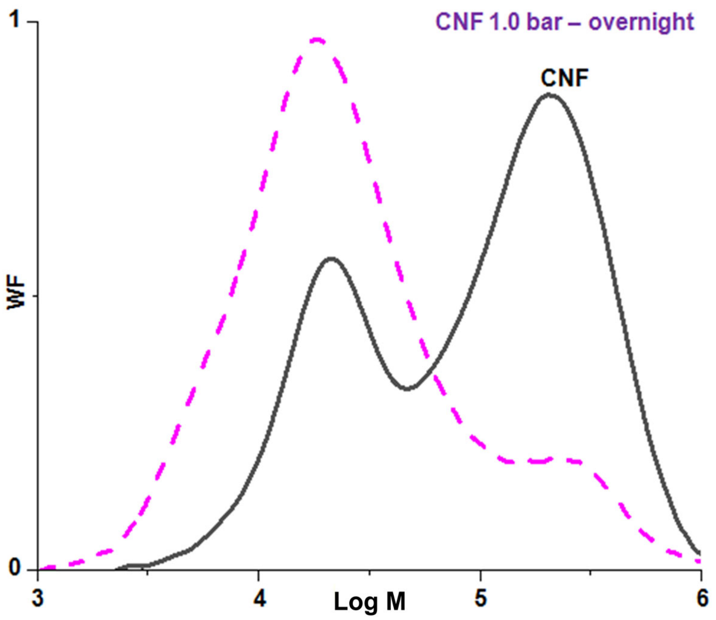

Figure S5. M distributions curves for the initial CNF aerogel and CNF aerogel hydrolyzed at 1.0 bar - overnight.

Figure S5 shows how overnight treatment of increased HCl (g) pressure led to intensive reduction of the unhydrolyzed CNF fraction, while the DP value dropped to 377. That corresponds to a further 33% decrease compared to the 1.0 bar - 30 min. hydrolyzed CNF sample.

1.4 X-Ray diffraction pattern (XRD)

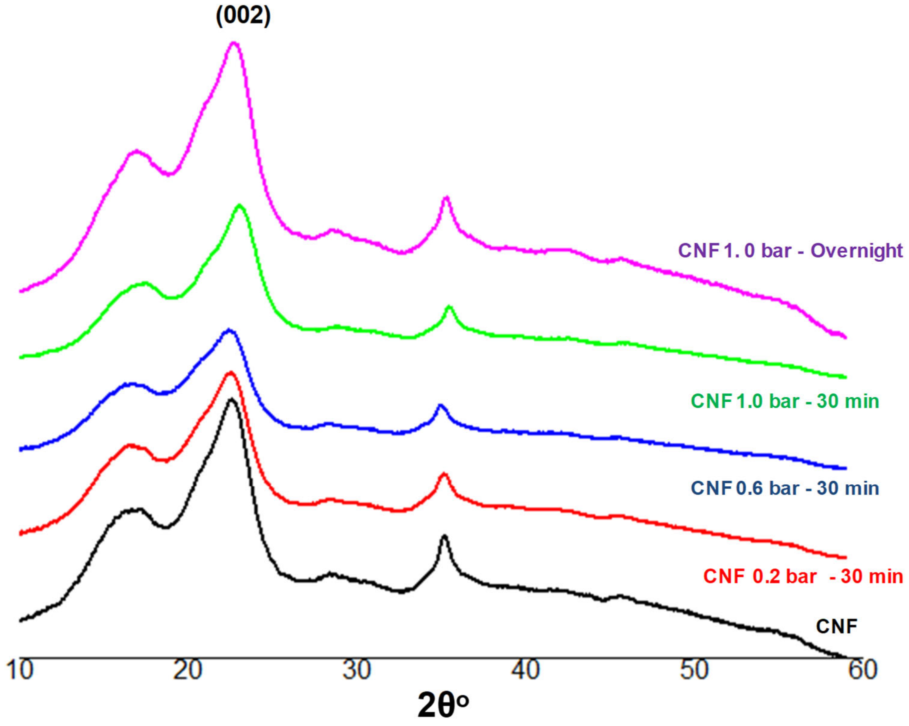

Figure S6. XRD pattern for the CNF and the hydrolyzed CNF aerogels.

Table S1. Peak position (200), crystallite average size as calculated by the 200 peak width by Scherrer equation and Full Peak Width at Half Maximum (FWHM) for the CNF hydrolyzed aerogels.

Sample	Peak Position (2θ°)	Crystallite size (nm)	FWHM
CNF	22.73	4.2	1.9873
CNF 0.2 bar – 30 min	22.76	4.3	1.965
CNF 0.6 bar – 30 min	22.76	4.3	1.9816
CNF 1.0 bar – 30 min	22.77	4.3	1.9361

1.5 Height Profile Images

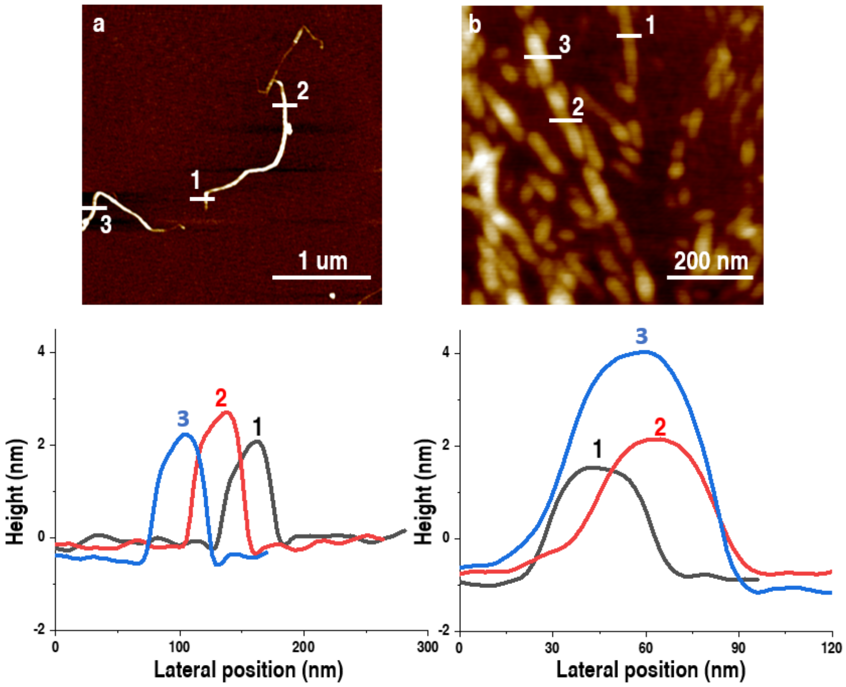

Figure S7. Height profile AFM images for the unhydrolyzed CNF (a) and for the 1.0 bar – 30 min hydrolyzed sample (b).

2. Additional calculations.

The molar mass distribution (Figure 6, Main article) derived from the length distribution for the CNF 1.0 bar sample, was extracted accordingly: Out of the length values (Figure 4b, Main article), the corresponding molar masses were calculated ($Length \times 2 \times 162$), considering that a DP value of 2 for cellulose corresponds to a length of 1.013 nm in a cellulose I crystal[5] and that the M_W of the anhydroglucose unit is 162 g/mol. The values were transformed into logarithmic scale and multiplied with the corresponding count values from the length histogram. The final length distribution (LD) extracted M histogram was ascribed by plotting the logarithmic values of the calculated M in the abscissa and the extracted count values (normalized) in the ordinate. Cellulose average crystallite sizes (Table 1, Supporting Info) were calculated through Scherrer's equation($\tau = k\,\lambda/\beta\, cos\theta$), while the line broadening value (β) was set at 0.3° and the shape factor (k) at 0.9.

References

1. Sluiter, A.; Hames, B.; Ruiz, R.; Scarlata, C.; Sluiter, J.; Templeton, D.; Crocker, D. *Determination of Structural Carbohydrates and Lignin in Biomass.* Technical Report for National Renewable Energy Laboratory: US, January 2008.

2. Borrega, M.; Carrasco, C. S.; Pranovich, A.; Sixta, H. Hot Water Treatment of Hardwood Kraft Pulp Produces High-Purity Cellulose and Polymeric Xylan. *Cellulose* **2017,** *24,* 5133-5145.

3. Lyczakowski, J. J.; Wicher, K. B.; Terrett, O. M.; Blanc, N. F.; Yu, X.; Brown, D.; Krogh, B. R. M. K.; Dupree, P.; Wicher, M. B. Removal of Glucuronic Acid from Xylan is a Strategy to Improve the Conversion of Plant Biomass to Sugars for Bioenergy. Biotechnol. **2017,** *10,* 1-11.

4. Edgar, C. D.; Grey, D. G. Smooth Model Cellulose I Surfaces from Nanocrystal Suspensions. *Cellulose* **2003,** *10,* 299-306.

5. Nishiyama, Y.; Sugiyama, J.; Chanzy, H.; Langan, P. Crystal Structure and Hydrogen Bonding System in Cellulose Iα from Synchrotron X-ray and Neutron Fiber Diffraction. *J. Am. Chem. Soc.* **2003,** *47,* 14300-14306.
